# Determinants of mortality risk in older adults from the ELSIA study: a prospective cohort study

**DOI:** 10.1590/1516-3180.2024.0402.R1.24032025

**Published:** 2025-10-06

**Authors:** Lucas Lima Galvão, Douglas de Assis Teles Santos, Claudio Andre Barbosa de Lira, Jair Sindra Virtuoso, Sheilla Tribess, Ricardo Borges Viana, Anne Sulivan Lopes da Silva Reis, Katja Weiss, Beat Knechtle, Rodrigo Luiz Vancini

**Affiliations:** I Physical Education Professional, Postgraduate student, Physical Education Sport Center, Universidade Federal do Espírito Santo (UFES), Vitória (ES), Brazil.; II Physical Education Professional, Assistant Professor, Department of Education, College of Physical Education, Universidade do Estado da Bahia (UNEB), Teixeira de Freitas (BA), Brazil.; III Associate Professor, Faculty of Physical Education and Dance, Universidade Federal de Goiás (UFG), Goiânia (GO), Brazil.; IV Physical Education Professional, Associate Professor, Department of Sport Sciences, Universidade Federal do Triângulo Mineiro (UFTM), Uberaba (MG), Brazil.; V Physical Education Professional, Associate Professor, Coordinator, Postgraduate Course in Physical Education, Department of Sport Sciences, Universidade Federal do Triângulo Mineiro (UFTM), Uberaba (MG), Brazil.; VI Physical Education Professional, Adjunct Professor, Institute of Physical Education and Sports, Universidade Federal do Ceará (UFC), Fortaleza (CE), Brazil.; VII Physical Education Professional, Postgraduate student, Physical Education Sport Center, Universidade Federal do Espírito Santo (UFES), Vitória (ES), Brazil.; VIII Professor, Researcher, Institute of Primary Care, University of Zurich, Zurich, Switzerland; Physician, Medbase St. Gallen Am Vadianplatz, St. Gallen, Switzerland.; IX Professor, Institute of Primary Care, University of Zurich, Zurich, Switzerland; Physician, Medbase St. Gallen Am Vadianplatz, St. Gallen, Switzerland.; X Associate Professor, Physical Education Sport Center, Universidade Federal do Espírito Santo (UFES), Vitória (ES), Brazil.

**Keywords:** Epidemiology., Health., Aging., Mortality., Public Health., Cohort., Physical activity., Brazil.

## Abstract

**BACKGROUND:**

This study investigated factors that may determine longevity in older adults, aiming to prolong their life expectancy and improve projections from before the coronavirus disease 2019 pandemic.

**OBJECTIVE:**

To identify risk factors for mortality in older Brazilian adults.

**DESIGN AND SETTING:**

A prospective cohort study, part of the *Estudo Longitudinal de Saúde do Idoso de Alcobaça*.

**METHODS:**

This study included 332 older adults of both sexes who were followed up for over five years (2015–2020). Vital status was determined via telephone follow-up, information provided by family members, and death certificates. To identify the sociodemographic, health, functional, and behavioral factors associated with mortality risk among older adults, Cox proportional hazards regression was used to estimate hazard ratios (HRs) and 95% confidence intervals (CIs).

**RESULTS:**

The risk factors for mortality among older adults included the number of people living with them (HR = 1.22; 95%CI = 1.07–1.38) and the number of prescribed drugs (HR = 1.15; 95%CI = 1.00–1.32). Factors associated with a lower risk of mortality were greater time spent in physical activity (HR = 0.99; 95%CI = 0.90–0.99) and greater hip circumference (HR = 0.95; 95%CI = 0.31–0.99).

**CONCLUSIONS:**

Sociodemographic, health, functional, and behavioral factors are determinants of mortality risk among older adults. Regular screening of the older adult population should be conducted to assess their general health status, allowing for more appropriate interventions to increase their quality of life and improve aging.

## INTRODUCTION

 The global older adult population is rapidly increasing, a trend that, coupled with consistent declines in birth rates, has shifted the global age pyramid.^1^ Projections indicate that the older adult population will triple by 2050, representing approximately 16% of the global population.^1^ In Brazil, the number of older adults is expected to grow by 56% over a 12-year period (2010-2022), with the median age increasing by six years to reach 35 years.^2^

 Conversely, the coronavirus disease 2019 (COVID-19) pandemic led to a significant number of deaths, particularly among older adults aged ≥ 60 years and individuals with pre-existing medical conditions, disrupting previously established projections.^3,4^ This resulted in a reduced life expectancy at birth in several countries,^5^ with some, including Brazil, experiencing mortality rates exceeding 50%.^6^

 Among the factors affecting mortality risk in older adults, sociodemographic factors such as age, marital status, and family structure are particularly significant.^7,8^ Identifying and explaining sex differences in mortality risk by marital status is crucial, as the impact of living alone versus living with a partner can differ between males and females.^8^ Health-related aspects, such as the emergence and progression of chronic diseases^9^ or the number of drugs ingested,^10-12^ also play a role in mortality risk by affecting metabolism and reducing longevity. Functional aspects, such as physical performance and independence, are additional factors that can affect mortality risk.^13,14^ Behavioral factors, including regular physical activity and time spent in sedentary behaviors, also contribute to mortality risk.^15 -18^ These elements have been identified as determinants of mortality risk and are instrumental in understanding the complex interactions between lifestyle and health outcomes in older adults.^15-18^

 Consequently, identifying factors that may serve as determinants of longevity in older adults is essential. Avoiding such risk factors and encouraging protective factors are important for extending life expectancy and improving projections established before the emergence of the COVID-19 pandemic,^3,4^ particularly among populations with low developmental indices. 

## OBJECTIVE

 This study aimed to identify the risk factors for mortality among participants of the *Estudo Longitudinal de Saúde do Idoso de Alcobaça* (ELSIA). We hypothesized that sociodemographic, health, functional, and behavioral factors may determine mortality risk among older adults, thereby affecting their longevity. 

## METHODS

### Ethical procedures

 This study was approved by the Human Research Ethics Committee of the Universidade Federal do Triângulo Mineiro (Ordinance 966.983/2015, February 27, 2015) and the Universidade do Estado da Bahia (Ordinance 3.471.114/2020, July 26, 2019). All protocols and procedures were conducted in accordance with the principles of the Declaration of Helsinki and Resolution No. 466/12 of the Brazilian Ministry of Health. All participants provided signed informed consent prior to participation. 

### Study design and participants

 This study is an excerpt from the ELSIA study, a prospective population-based cohort study that used survey methods and physical performance tests. This study adhered to the Strengthening the Reporting of Observational Studies in Epidemiology (STROBE) guidelines. 

 The baseline study, conducted between June and September 2015, included a sample of 473 older adults of both sexes, aged ≥ 60 years, who were registered with the family health strategy in the municipality of Alcobaça, a program aimed at enhancing and solidifying Primary Care in Brazil, following the principles of the Unified Health System. This information has been previously described by Galvão et al.^19^ The follow-up continued for five years, between January and February 2020, with older adults invited to participate in the second wave of the research. 

 The inclusion criteria were signing an informed consent form, being registered with the family health strategy, and residing in the community. The exclusion criteria included scores ≤11 points on the Mini-Mental State Examination, indicating cognitive impairment;^20,21^ experiencing severe difficulty with visual and/or hearing acuity; requiring the use of a wheelchair; having severe sequelae from a cerebrovascular accident with localized loss of strength; or having a terminal illness. A comprehensive overview of the participant descriptions, attrition rates, recruitment strategies, and other variables investigated can be found in previous studies.^22^

### Mortality

 Vital status was determined through telephone monitoring, information provided by family members, including death certificates; data obtained from the Civil Registry Office of Natural Persons of Alcobaça; and public consultations on the website of the Court of Justice of the State of Bahia. To calculate the survival time, the follow-up period was defined as the time elapsed from the beginning of the survey until death, censoring, or the end of the second phase (February 29, 2020). 

### Sociodemographic aspects

 The assessed sociodemographic characteristics included age, income, sex (male or female), which was used as an adjustment factor in the model, and the number of people living in the same house as the participant. 

### Health aspects

 The evaluated health aspects included the total number of hospitalizations and falls recorded in the 12 months preceding the assessment, the number of prescribed drugs, and the presence of diseases (< 2 and ≥ 2), assessed from a list of diseases proposed by the World Health Organization in the 10 ^th^ revision of the International Classification of Diseases.^23^

### Functional and behavioral aspects

 Body mass was measured using a Wiso digital scale (W721) with a capacity of 180 kg and a precision of 0.100 g and 0.1 cm. The circumferences of the arm, thigh, and calf were also evaluated using standardized measurements on the participants’ right side. Hip circumference was measured at the point of the greatest prominence in the region. All evaluations were performed using a flexible and inelastic measuring tape (Lange-TBW, Cambridge, Massachusetts, United States) 2 m in length, graduated in centimeters, and subdivided into millimeters.^24^

 Disability in basic daily living activities was evaluated using the Katz scale, which has been adapted for the older Brazilian adult population.^25^ The scale ranges from 0 to 12 points. For the analyses, the participants were dichotomized into independent (0 points) and dependent (≥ 1 point). Disability in instrumental activities of daily living was assessed using the Lawton and Brody Scale, which has also been adapted for older adult populations in Brazil.^26^ The scale ranges from 0 to 14 points. For analyses, participants were dichotomized into independent (≥ 11 points) and dependent (< 11 points) groups. 

 Physical function was assessed using the Fullerton Functional Fitness Test,^27^ with percentile distributions adopted as cutoff points.^28^ The muscular strengths of the upper and lower limbs were evaluated using two tests: the elbow flexion test, using a 2 kg load for females and a 3 kg load for males, which involved older adults performing as many elbow flexions as possible within 30 seconds. The chair sit-and-stand test required participants to sit down and stand up from a chair without hand support as many times as possible within 30 s. The number of completed repetitions was recorded to analyze strength. For categorical data analysis, percentiles ≤ P25 were considered indicative of lower strength, whereas percentiles > P25 indicated higher strength. 

 A stationary walking test was used to evaluate aerobic endurance. The older adult participants were instructed to raise their knees to a predetermined height as many times as possible within 2 min. For the analysis of categorical data, the participants were classified based on percentiles, with ≤ P25 indicating lower endurance and > P25 indicating higher endurance. 

 Agility and dynamic balance were assessed using the 2.44-meter timed up and go test. The test began with the participants seated; when instructed, they walked as quickly as possible to a cone, circled it, and returned to their seats. For the analysis of categorical data, the participants were classified based on percentiles, with ≤ P75 representing slower times and > P75 indicating faster times. 

 Physical activity (PA) and exposure time to sedentary behavior were assessed using the International Physical Activity Questionnaire.^29,30^ PA was evaluated based on moderate-to-vigorous-intensity activity (MVPA) measured in 10-minute bouts. Sedentary behavior was defined as the time spent sitting, lying, or reclining during waking hours. This was assessed using questions about sitting time on a typical weekday ("How much total time do you spend sitting during a weekday?") and a typical weekend day ("How much total time do you spend sitting during a weekend day?"). The total time spent sitting in minutes per day was calculated from the weighted average of the sitting time on weekdays and weekends: Total time spent sitting = [(sitting time on a weekday × 5) + (sitting time on a weekend day × 2)]/7.^31^

 Frailty syndrome was assessed using the adapted version of Fried et al.’s frailty phenotype,^32^ which considers four components: unintentional weight loss, exhaustion, decreased muscle strength, and slow gait speed. Unintentional weight loss was assessed by asking, "In the last year, have you lost more than 10 pounds unintentionally (i.e., without dieting or exercising)?" Participants who answered affirmatively were considered to meet this criterion for frailty. Exhaustion was identified through two questions from the Geriatric Depression Scale (GDS-15), translated and validated for the Brazilian population:^33^ "Did you stop doing many of your activities and interests?" and "Do you feel full of energy?" A positive answer to the first question and/or a negative answer to the second question indicated exhaustion or fatigue, thus meeting this criterion for frailty. 

 A decrease in muscle strength was assessed based on handgrip strength measured in kilograms of force (kgf) using an SAEHAN hydraulic dynamometer (Saehan Corporation SH5001, Korea). The participants were instructed to remain standing with their elbows extended, press the handle of the dynamometer with the highest force possible using their dominant hand, and hold it for 6 s (self-reported by the participant). Three measurements were obtained, with a 1-minute recovery time between attempts. The best performance was used for the analysis.^32^

 Walking speed was measured using the 4.57-meter walk test, adjusted for sex and height.^32^ Older adults who scored above the cutoff point for the walking test and those who were unable to perform it because of physical limitations met this criterion for frailty. 

 Frailty was measured dichotomously: older adults who scored 0, 1, or 2 points were classified as robust, while those who scored ≥ 3 points were classified as frail. 

### Statistical analysis

 Statistical analyses were conducted using IBM SPSS Statistics for Windows, version 26.0 (IBM Corp., Armonk, New York, United States), and JASP version 0.18.3.0 (University of Amsterdam, Amsterdam, Netherlands). Data normality was assessed using the Kolmogorov–Smirnov test. 

 Descriptive statistics, including absolute and relative frequencies, medians, and interquartile ranges (IQRs), were used to examine the samples. To compare participants based on their vital status, the chi-square and Mann–Whitney U tests were used for categorical and continuous variables, respectively. Median differences expressed as Δ were calculated for continuous variables according to the vital status. Effect sizes were calculated using rank biserial correlation (rB) and the respective 95% confidence intervals (CIs), with values trivial (< 0.10), small (0.10–0.29), medium (0.30–0.49), or large (≥ 0.5).^34^

 To identify the risk factors for mortality, crude and multivariate analyses were performed with hazard ratio (HR) estimates using Cox proportional hazards regression. Bivariate models were constructed for each independent and response variable (mortality). Variables with P < 0.05 were considered candidates for inclusion in the hierarchical multivariable model. Variables were introduced into blocks in the model, which was controlled for age, sex, and income. Block 1 contained sociodemographic aspects, such as the number of people living in a household. Block 2 included health aspects, such as the number of hospitalizations, falls, and drug use. Block 3 encompassed functional aspects (basic activities of daily living, instrumental activities of daily living, aerobic endurance, upper limb strength, lower limb strength, agility, dynamic balance, and frailty syndrome), anthropometric aspects (body mass, arm, hip, thigh, and calf circumferences), and behavioral aspects (PA and sedentary behavior). A significance level of 5% and 95%CI were used to calculate HRs. 

## RESULTS

**Figure 1:**
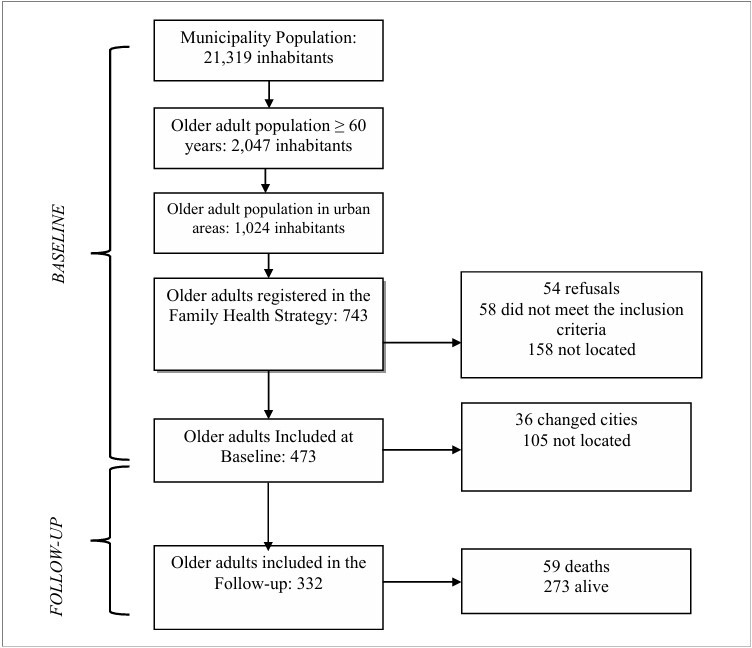
Flowchart of the study participants.

 During the follow-up of the 473 older adults included in the baseline study, 105 could not be located, and 36 had moved to other cities, resulting in the exclusion of 141 participants. Of the 332 participants who returned, 59 died and 273 were alive. The second phase occurred between January and February 2020. The characteristics of older adults are shown in **Figure 1**; additional information is described in previous studies.^22^

 Among the participants included in the present study, 121 were male (36.4%), and 23 (19%) died during the follow-up period. Of the 211 female participants (63.6%), 36 (17.1%) had died. The mean age of the living participants was 69.7 ± 7.6 years, whereas the mean age of those who died was 76.9 ± 9.6 years. The average income of the surviving and deceased participants was 2,089 ± 3,492 Brazilian reais and 2,089 ±1,949 Brazilian reais, respectively. 

**Table 1** describes the categorical variables for the vital status of the study participants. Among the participants who died during the study, the highest prevalence was observed in individuals with more diseases, those who were dependent, and those with worse performance on the physical tests. Although a higher absolute number of deaths occurred among non-frail participants, the percentage of deaths was significantly higher in the frail group. 

**Table 1:** Comparisons of qualitative variables among *Estudo Longitudinal de Saúde do Idoso de Alcobaça* (ELSIA) study participants according to vital status

**Variables**		**Alive**	**Deceased**	**P**
		**n (%)**	**n (%)**	
**Diseases**				
	< 2	145 (87.3)	21 (12.7)	0.015
	≥ 2	128 (77.1)	38 (22.9)	
**Basic activities of daily living**				
	Independent	222 (85.4)	38 (14.6)	0.004
	Dependent	51 (70.8)	21 (29.2)	
**Instrumental activities of daily living**				
	Independent	194 (89.4)	23 (10.6)	< 0.001
	Dependent	79 (68.7)	36 (31.3)	
**Aerobic endurance**				
	Good	205 (89.5)	24 (10.5)	< 0.001
	Moderate or poor	55 (67.9)	26 (32.1)	
**Upper limb strength**				
	Good	193 (88.9)	24 (11.1)	< 0.001
	Moderate or poor	70 (70.7)	29 (29.3)	
**Lower limb strength**				
	Good	191 (89.7)	22 (10.3)	< 0.001
	Moderate or poor	74 (69.8)	32 (30.2)	
**Agility and dynamic balance**				
	Good	207 (89.2)	25 (10.8)	< 0.001
	Moderate or poor	59 (67.8)	28 (32.2)	
**Frailty syndrome**				
	Not fragile	249 (86.2)	40 (13.8)	< 0.001
	Fragile	17 (54.8)	14 (45.2)	

 The quantitative data and their relationships with vital status are presented in **Table 2**. Except for the number of hospitalizations and falls, all other variables differed according to the vital status. The observed effect sizes ranged from trivial (d = -0.094) to medium (d = 0.401). 

**Table 2:** Comparison of quantitative variables among Estudo Longitudinal de Saúde do Idoso de Alcobaça (ELSIA) study participants according to vital status

**Variables**	**Median (IQR)**		**Δ**	**P**	**rB (95% CI)**
	**Alive**	**Deceased**			
**Number of people**	3.0 (2.0)	3.0 (3.0)	–0.93	0.088	-0.137 (-2.29 to 0.02)
**Number hospitalization**	3.0 (2.0)	3.0 (3.0)	–0.30	0.086	-0.094 (-0.25 to 0.06)
**Number of falls**	0.0 (0.0)	0.0 (0.0)	–0.39	0.061	-0.125 (-0.28 to 0.03)
**Number of drugs**	0.0 (1.0)	0.0 (1.0)	–1.15	< 0.001	-0.291 (-0.43 to -0.13)
**Body mass (kg)**	2.0 (3.0)	3.0 (3.0)	5.79	0.0021	0.195 (0.03–0.34)
**Arm circumference (cm)**	66.3 (19.3)	63.0 (21.0)	2.20	< 0.001	0.309 (0.15–0.45)
**Hip circumference (cm)**	29.6 (5.3)	27.5 (6.0)	4.62	< 0.001	0.285 (0.12–0.42)
**Thigh circumference (cm)**	99.0 (12.8)	93.7 (11.8)	4.26	< 0.001	0.323 (0.16–0.46)
**Calf circumference (cm)**	34.9 (5.0)	33.0 (4.6)	2.08	< 0.001	0.326 (0.17–0.46)
**Physical activity (min/week)**	200.0 (475.0)	10.0 (205.0)	260.84	< 0.001	0.401 (0.25–0.52)
**Sedentary behavior (min/day)**	395.7 (202.1)	480.7 (237.2)	–84.36	< 0.001	-0.275 (-0.41 to -0.11)

IQR = interquartile range; Δ = medium difference; CI = confidence interval; rB = rank biserial correlation.

 The risk factors analyzed using Cox regression analysis are presented in **Table 3**. After adjusting for sex, age, and income, the identified risk factors for mortality were the number of people with whom the older adult lived (HR = 1.22; 95%CI = 1.071.38) and the number of prescribed drugs consumed (HR = 1.15; 95%CI = 1.00–1.32). Conversely, greater time spent in PA (HR = 0.99; 95%CI = 0.90–0.99) and greater hip circumference 

**Table 3:** Risk factors for mortality among *Estudo Longitudinal de Saúde do Idoso de Alcobaça* (ELSIA) study participants

Variables		**Raw analysis**		**Multivariable analysis**	
		**HR (95% CI)**	**P**	**HR (95% CI)**	**P**
**Block 1–Sociodemographic aspects**					
**Number of people**		1.19 (1.08–1.31)	< 0.001	1.22 (1.07–1.38)	0.002
**Block 2–Health aspects**					
**Number of hospitalizations**		1.37 (1.11–1.69)	0.003	1.19 (0.92–1.54)	0.251
**Number of falls**		1.15 (1.01–1.31)	0.025	1.19 (0.85–1.78)	0.181
**Number of drugs**		1.16 (1.07–1.27)	< 0.001	1.15 (1.01–1.32)	0.041
**Block 3–Functional, anthropometric, and behavioral aspects**					
**Body mass**		0.97 (0.95–0.99)	0.004	1.04 (0.99–1.09)	0.055
**Arm circumference**		0.88 (0.83–0.95)	0.001	0.97 (0.85–1.13)	0.700
**Hip circumference**		0.97 (0.95–0.99)	0.004	0.95 (0.31–0.99)	0.017
**Thigh circumference**		0.91 (0.87–0.95)	< 0.001	0.98 (0.90–1.08)	0.772
**Calf circumference**		0.86 (0.79–0.93)	< 0.001	0.88 (0.76–1.01)	0.077
**Basic activities of daily living**			0.002		0.826
	Independent	1		1	
	Dependent	2.28 (1.33–3.91)		0.91 (0.42–1.98)	
**Instrumental activities of daily living**			< 0.001		0.645
	Independent	1		1	
	Dependent	3.28 (1.94–5.56)		0.82 (0.36–1.87)	
**Physical activity**		0.99 (0.99–0.99)	0.001	0.99 (0.98–0.99)	0.045
**Sedentary behavior**		1.00 (0.99–1.00)	< 0.001	0.99 (0.99–1.00)	0.618
**Aerobic endurance**			< 0.001		0.250
	Good	1		1	
	Moderate or poor	3.72 (2.12–6.52)		1.57 (0.72–3.42)	
**Upper limb strength**			< 0.001		0.737
	Good	1		1	
	Moderate or poor	2.96 (1.71–5.10)		1.13 (0.54–2.38)	
**Lower limb strength**			< 0.001		0.746
	Good	1		1	
	Moderate or poor	3.50 (2.02–6.07)		1.14 (0.50–2.57)	
**Agility and dynamic balance**			< 0.001		0.097
	Good	1		1	
	Moderate or poor	3.69 (2.14–6.37)		2.10 (0.87–5.05)	
**Frailty syndrome**			< 0.001		0.814
	Not fragile	1		1	
	Fragile	3.04 (1.77–5.22)		0.91 (0.42–1.98)	

Adjusted for sex, age, income, and the presence of diseases. HR = hazard ratio; CI = confidence interval.

## DISCUSSION

 This study aimed to identify the risk factors for mortality among the ELSIA participants. The identified risk factors included the total number of people and older adults living with the participants and the total number of medications consumed. Additionally, hip circumference and PA were also associated with mortality. These findings confirmed our hypothesis that sociodemographic, health, functional, and behavioral factors can determine mortality risk among older adults, thus affecting their longevity. 

 Among the sociodemographic conditions associated with mortality among older adults, the only variable that remained significantly associated with the risk of mortality, even after model adjustment, was the number of people living in the same household, with a 22% increase in mortality risk. These findings differ from those reported previously, demonstrating that living with a partner, can lower the risk of death compared to living alone.^7,8,35^

 Being married can reduce mortality risk, depending on sex and age group.^7,8^ A study investigating the role of living arrangements on mortality risk among approximately 54,000 European older adults aged ≥ 50 years observed higher mortality risks among individuals who lived alone. Men who lived with people other than their partners exhibited a higher risk of mortality compared with those who lived with a partner.^7^ This finding aligns with those of other studies reporting that divorced or single males tend to have worse health outcomes compared with single females, suggesting that marital status may have a protective effect against mortality risk and may be linked to psychological factors, although the quality of relationships must also be considered.^35^

 A cohort study conducted among older Japanese adults investigated the associations between family relationships in various domestic contexts, including the functional dimensions of support provided and received from children. The study identified a higher mortality rate among participants who received more support from their children (HR = 1.07), while those who provided more support to their children had a lower mortality rate (HR = 0.88) despite the unique social context in Japan.^14^ Similarly, we hypothesized that the number of people living in the same house may be a risk factor based on the concept of independence, as a greater number of individuals living with older adults may hinder their ability to carry out basic and routine activities because of concerns and overprotection from younger family members, including children and grandchildren. 

 Physical and functional independence is crucial for the better quality of life and longevity of older adults. Galvão et al.^13^ identified a mediating factor between functional performance and basic activities of daily living based on PA practice for survival time in an older adult population, suggesting that better scores on these variables are associated with greater longevity. 

 In this study, the number of medications was associated with a 15% increase in mortality risk, reflecting the impact of polypharmacy, commonly defined as the use of multiple medications.^10^ Individuals with multiple comorbidities often take several medications concurrently, which can lead to inappropriate prescribing and various medication-related issues, including falls, fractures, kidney failure, increased frailty risk, decreased quality of life, hospitalizations, and, ultimately, increased mortality risk.^11^ In their investigation of > 3 million older adults aged ≥ 65 years who continuously used at least one medication, Chang et al.^10^ observed linear associations between mortality risk and hospitalizations among older adults, even in subgroup analyses by sex and age. Another study involving 1,258 older adult aged ≥ 60 years reported a 77.2% survival rate among participants with polypharmacy (≥ 5 medications), compared with 85.5% among those taking < 4 medications.^36^

 Although isolating the number of medications as an independent factor is difficult, as it often correlates with the number of pre-existing conditions, polypharmacy itself poses potential risks, even though medications are prescribed to improve health.^9, 10^ The findings of this study underscore the risks of polypharmacy and the critical need for careful and appropriate prescribing practices, along with strategies to minimize polypharmacy in the geriatric population.^10^ Alternatives, such as non-pharmacological approaches, including regular PA, should be prioritized whenever possible to mitigate these risks. 

 PA is a modifiable behavioral factor that is crucial for longevity. In the present study, PA was found to be a protective factor against mortality (HR = 0.99). Although this value appears low, regular exercise reduces the risk of mortality in older adults,^16,37^ regardless of sedentary time.^16,17^ Ekelund et al.^17^ demonstrated that regularly practicing MVPA for 60–75 min/day reduces the risk of mortality, regardless of sedentary time. Similarly, Stamatakis et al.^16^ reported that ≥ 300 min of MVPA eliminated the mortality risks associated with sedentary time. 

 In the present study, hip circumference was a modifiable factor associated with reduced mortality risk. We observed a 5% lower risk of mortality among individuals with a larger hip circumference, consistent with other studies reporting inverse associations between hip circumference and mortality risk.^38^ For instance, a study of 10,767 participants reported that a smaller hip circumference was a risk factor for diabetes and coronary artery disease.^39^ However, a study that monitored changes in hip circumference over six years and assessed the risk of mortality observed that lower baseline hip circumference was a risk factor regardless of body mass index and waist circumference, but observed no associations with changes in hip circumference over time.^40^ These contrary results suggest the need for further studies to explore whether circumference measurement is the optimal method for cardiovascular disease screening. Additionally, normative values should be developed similar to those established for body mass index, waistto-hip ratio, and waist-to-height ratio to guide the interpretation of hip circumference and its association with various functional and physical outcomes. 

### Limitations, strengths, and practical applications

 Our study had some limitations, including the use of subjective measures that could introduce bias. However, this was mitigated through prior training of the data collection team, resulting in a sample loss of approximately 30%. Additionally, the model included only variables that showed an isolated association with 5% mortality. Other factors that may be associated with the outcomes were not evaluated. The strengths of this study include the use of a representative sample with five years of follow-up and a study city with a low human development index in a developing country. Additionally, identifying risk factors that may be crucial for improving the quality of life and longevity of the population is a significant advantage. Public policies should prioritize promoting regular PA, particularly among older adults, with practices oriented toward health development, physical, and functional independence. These policies should also aim to reduce drug consumption while consistently seeking to improve the quality of life and foster successful aging. 

## CONCLUSION

 Among the older adults included in this study, the determinants of mortality risk were the total number of people living in the same household and the total number of prescribed drugs. These findings suggest that drug prescriptions should be approached with caution in this population. Furthermore, promotion of healthier lifestyles and greater independence in daily activities should be prioritized. Conversely, greater hip circumference and increased PA were also inversely associated with mortality, suggesting that improving these factors could help reduce the risk of mortality. Regular screening of older adults is essential for monitoring their overall health status, identifying potential health issues early, and enabling timely and appropriate interventions. Such interventions can improve the quality of life and contribute to healthy aging. 
